# Supplementary material for: Non-canonical EGFR signaling promotes MAPK-dependent extrusion of epithelial cells
Source: J Cell Sci. 2025 Dec 12;138(23):jcs264173. doi: 10.1242/jcs.264173 (PMC12752489; doi:10.1242/jcs.264173)
Supplement: Supplementary information [file joces-138-264173-s1.pdf]

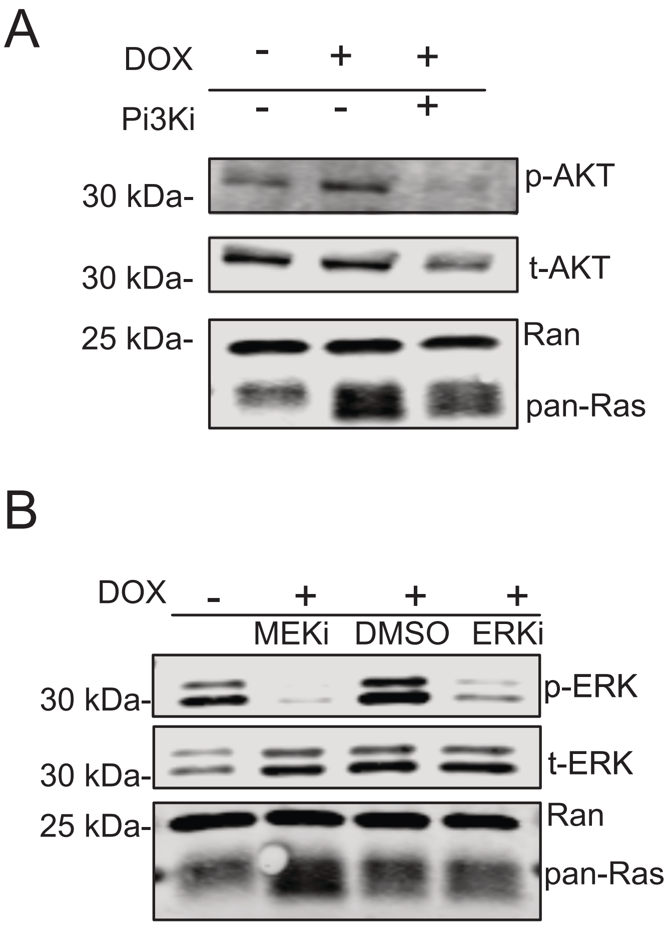

Fig. S1. Validation of inhibitors.

(A)PI3Ki (LY294002) inhibitor suppresses AKT phosphorylation.

(B) MEKi (U0126) and ERKi (SCH772984) effectively block ERK phosphorylation. (C) EGFRi (Erlotinib) reduces EGFR phosphorylation. Representative immunoblots of cell lysates from RasQ61L Eph4 cells +/- Dox.

Journal of Cell Science • Supplementary information

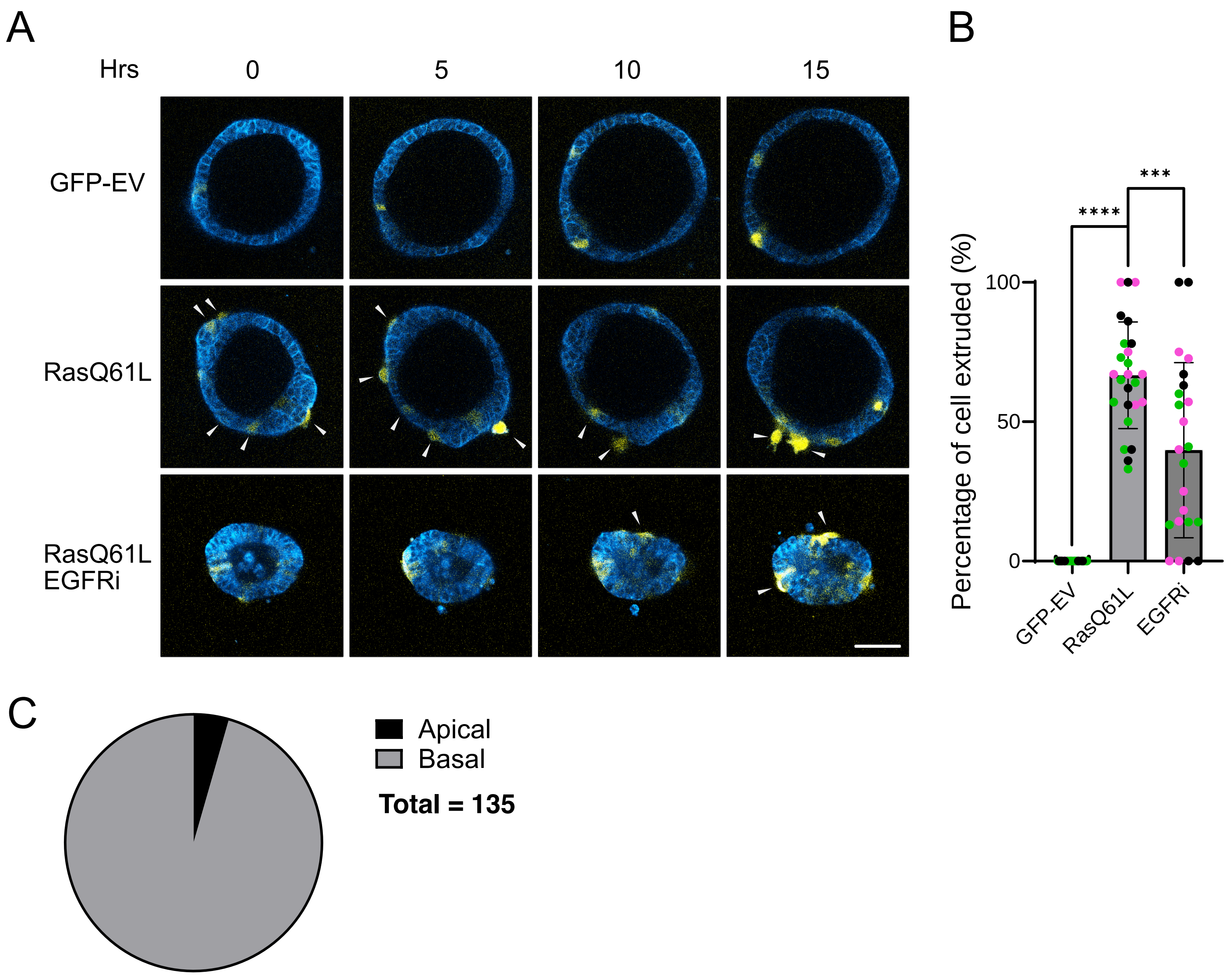

Fig. S2. Ras expression induces basal extrusion of primary mammary epithelial cells, which is suppressed by Erlotinib.

(A) Representative images of GFP-EV, RasQ61L GFP, and EGFRi (Erlotinib)-treated RasQ61L GFP cysts, showing Ras-stimulated basal extrusion and partial inhibition by Erlotinib. Time-lapse confocal imaging was performed over 15 hrs. Cell membranes are shown in blue (CellBrite), and GFP^+ cells in yellow. White arrows indicate extruded cells. Scale bar=50 µm.

(B) Quantification of extrusion events. The percentage of GFP^+ cells that extruded from the cyst was determined at the final imaging frame (15 hr). Statistical comparisons were made using unpaired parametric t-tests. GFP-EV vs RasQ61L: $p < 0.0001$; RasQ61L vs EGFRi: $p = 0.0006$. Bars show mean ± 1 SD (n = 3).

(C) Quantification of the direction of extrusion events in RasQ61L-expressing primary mammary epithelial cysts. Among a total of 135 extrusion events pooled from 3independent experiments (n=3), 129 were directed basally and 6 apically, indicating a predominantly basal extrusion phenotype.

Journal of Cell Science • Supplementary information

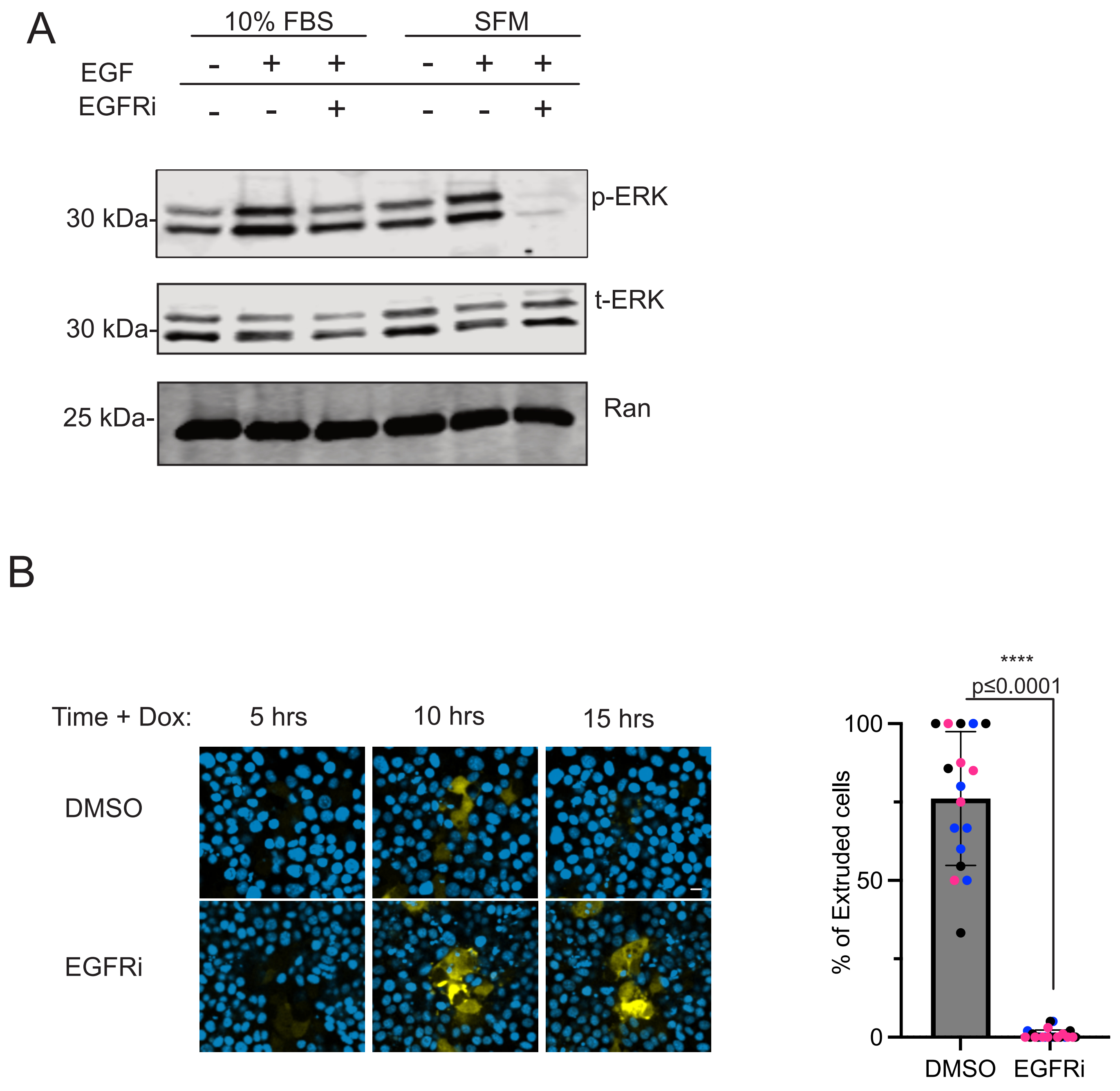

Fig. S3. Erlotinib blocks extrusion in the presence of serum, which stimulates ERK phosphorylation independently of EGFR.

(A) Immunoblot for ERK phosphorylation on lysates of Eph4 cells cultured either in medium with 10% FBS or in serum-free medium (SFM), +/- EGF and +/- Erlotinib to inhibit the EGFR. Note that Erlotinib has very little effect on ERK phosphorylation in FBS, but almost completely blocks ERK phosphorylation in SFM.

(B) Effect of EGFR inhibition on Ras(Q61L) extrusion in 10% FBS medium. WT cells were labeled with mCherry-H2B, which marks the nuclei (blue). Ras+ cells are marked by GFP after Dox induction (yellow). Note that despite the lack of effect on phospho-ERK levels, Erlotinib still blocks extrusion, suggesting that the role of EGFR in extrusion is through a noncanonical pathway independent of ERK phosphorylation. Bars represent mean +/- 1 SD (N=3). Groups were compared using a two-tailed unpaired t-test.

Journal of Cell Science • Supplementary information

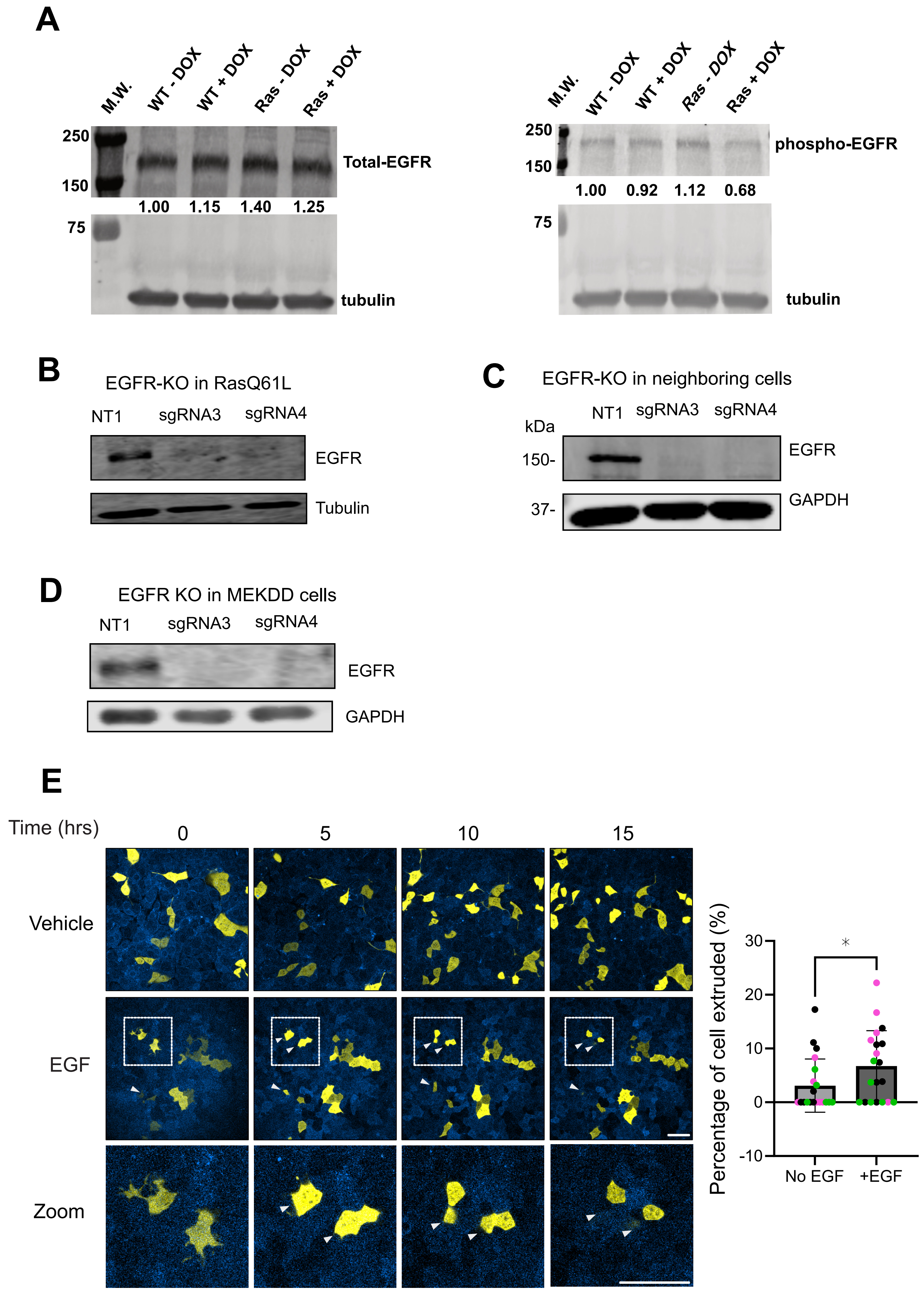

Journal of Cell Science • Supplementary information

Fig. S4. EGFR levels +/- Ras expression, knockout of EGFR in WT, Ras(Q61L) and MEKDD cells, and apical extrusion triggered by EGFR.

(A) Immunoblots of total EGFR and phospho-EGFR on lysates of WT Eph4 cells or cells that express Dox-inducible oncogenic Ras, +/- Doxycycline. Tubulin was used as a loading control. Numbers represent the mean levels of total EGFR or phospho-EGFR normalized to tubulin and to the WT cells -Dox. (B)

(B - D) Cas9-mediated knockout of EGFR using two distinct sgRNAs. Cell lysates were immunoblotted for EGFR, plus GAPDH or Tubulin as a loading control.

(E) WT cells (expressing EGFR) were labeled with mApple (yellow) and mixed at a ratio of 1:50 with cells deleted for EGFR and labeled with CSFE (carboxyfluorescein succinimidyl ester; blue), in serum-free medium. Ligand (EGF) was added at 20 ng/ml and cells were imaged for 15 hrs.

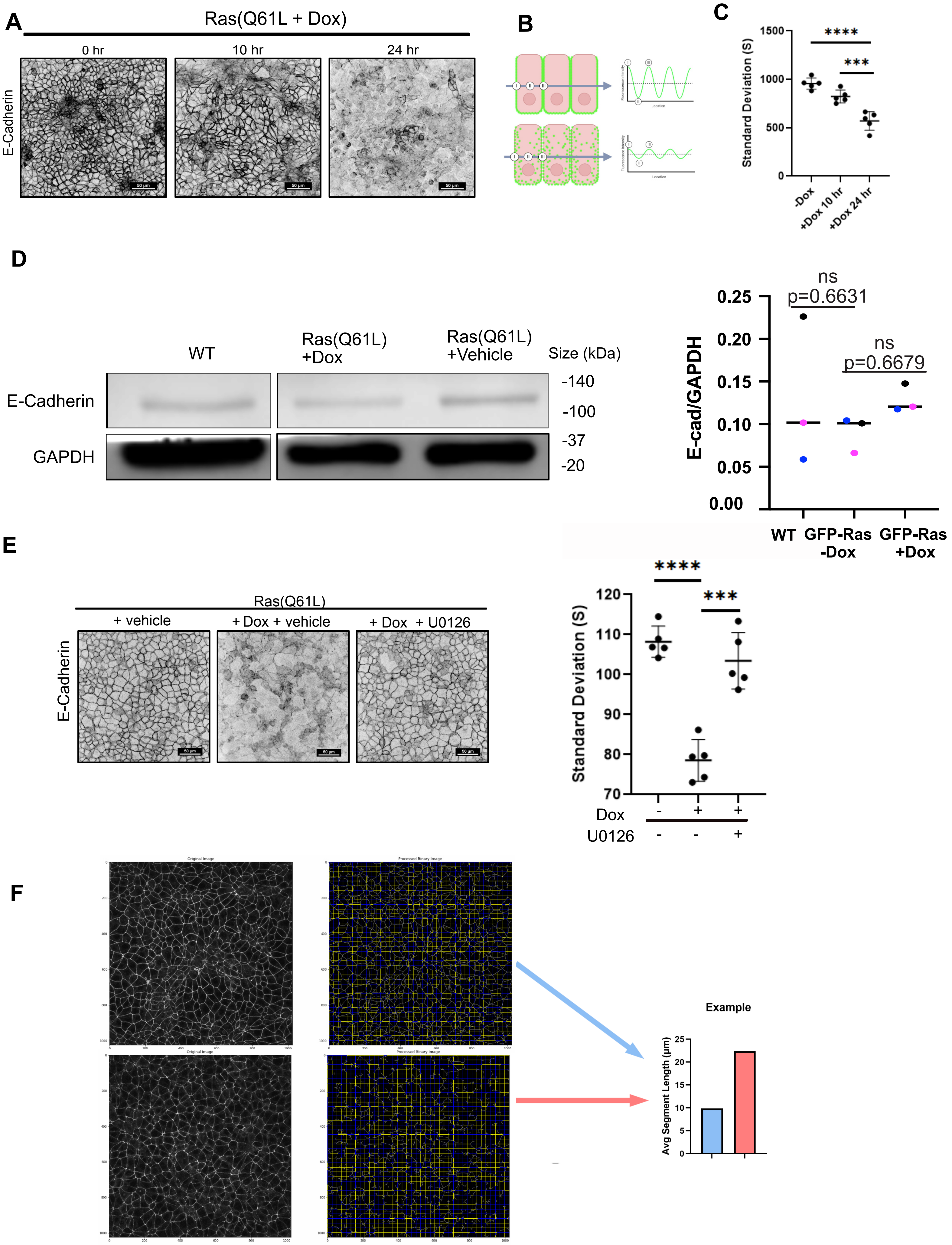

Journal of Cell Science • Supplementary information

Fig. S5. E-cadherin internalization driven by Ras(Q61L) expression.

(A) Eph4 cells were treated +/- Dox to induce oncogenic Ras and fixed at intervals for staining with anti E-cadherin antibody. Loss of E-cadherin from the adherens junctions is apparent by 24 hrs.

(B) Lines were drawn across multiple fields of view to measure the variance in staining intensity. Cells with intact junctions show high variance, as compared to those cells in which the E-cadherin has been internalized.

(C) Quantification is shown of variance across multiple fields of view.

(D) E-cadherin immunoblot on lysates from WT or Ras(Q61L) cells +/- Dox for 24 hrs. Blots were scanned to quantify band intensities. No significant difference was found between conditions (N=3 biological replicates) showing that the disappearance of E-cadherin from the junctions is not caused by degradation of protein. One-way ANOVA was used to test significance.

(E) IF images of Ras(Q61L)-expressing cells treated +/- Dox and MEK inhibitor U0126. E-cadherin localization was assessed to evaluate internalization in response to Ras activation. Quantification reflects measurements from multiple independent fields of view.

(F) Schematic of a robust and quantitative assay for junction integrity, applicable to videos of epithelial cell dynamics. For each video a grid of horizontal and vertical lines is drawn, and for each time point the length of each segment of the grid is determined between pixels of high intensity (top panels white in the figure). To avoid inaccuracies caused by differences in cell size, all measurements are performed on cells plated at the same density. To make segment lengths easily visible, they are shown in a different color (yellow or blue) after crossing a junction. As junctions dissolve, the segment lengths increase
(bottom panels), as illustrated in the example on the right.

Journal of Cell Science • Supplementary information

Table S1. Inhibitors and other reagents.

Name	Manufacturer	Concentration	Cat. Number
Erlotinib	Sigma Aldrich	10 μM	CDS022564
LY294002	Cell Signaling	10 μM	9901S
U0126	Tocris	10 μM	1144
SCH772984	Selleck Chemicals	10 μM	S7101
CellTrace CSFE	Invitrogen	5 μM	C34554
CellBrite	Biotium	1:1000	30108-T
Doxycycline	Sigma Aldrich	1 μg/ml	D5207

Table S2. Antibodies.

Name	Manufacturer	Cat. Number	Application	Dilution
Pan Ras	**Calbiochem**	**OP40**	**western**	**1:1000**
GFP	**homemade**		**western**	**1:1000**
Ran	**homemade**		**western**	**1:3000**
EGFR	**EMD Millipore**	**06-847**	**western**	**1:500**
PhosphoEGFR	**Cell Signaling**	**2234S**	**western**	**1:1000**
MAPK	**Cell Signaling**	**4696S**	**western**	**1:1000**
Phospho MAPK	**Cell Signaling**	**4370S**	**western**	**1:1000**
AKT	**Cell Signaling**	**4691**	**western**	**1:1000**
Phospho AKT	**Cell Signaling**	**4060**	**western**	**1:1000**
SOS1	**Santa Cruz**	**sc-11793**	**Western**	**1:500**
SOS2	**Santa Cruz**	**sc-258**	**western**	**1:500**
Tubulin	**MilliporeSigma**	**T9026**	**western**	**1:5000**
GAPDH	**Cell Signaling**	**2118S**	**Western**	**1:3000**
Alexa Fluor 680 donkey anti-mouse IgG (H+L)	**Invitrogen**	**# A10038**	**WB**	**1:10000**
Goat anti-Rabbit (H&L), DyLight 800 4X PEG conjugate	**Invitrogen**	**# SA535571**	**WB**	**1:10000**

Journal of Cell Science • Supplementary information

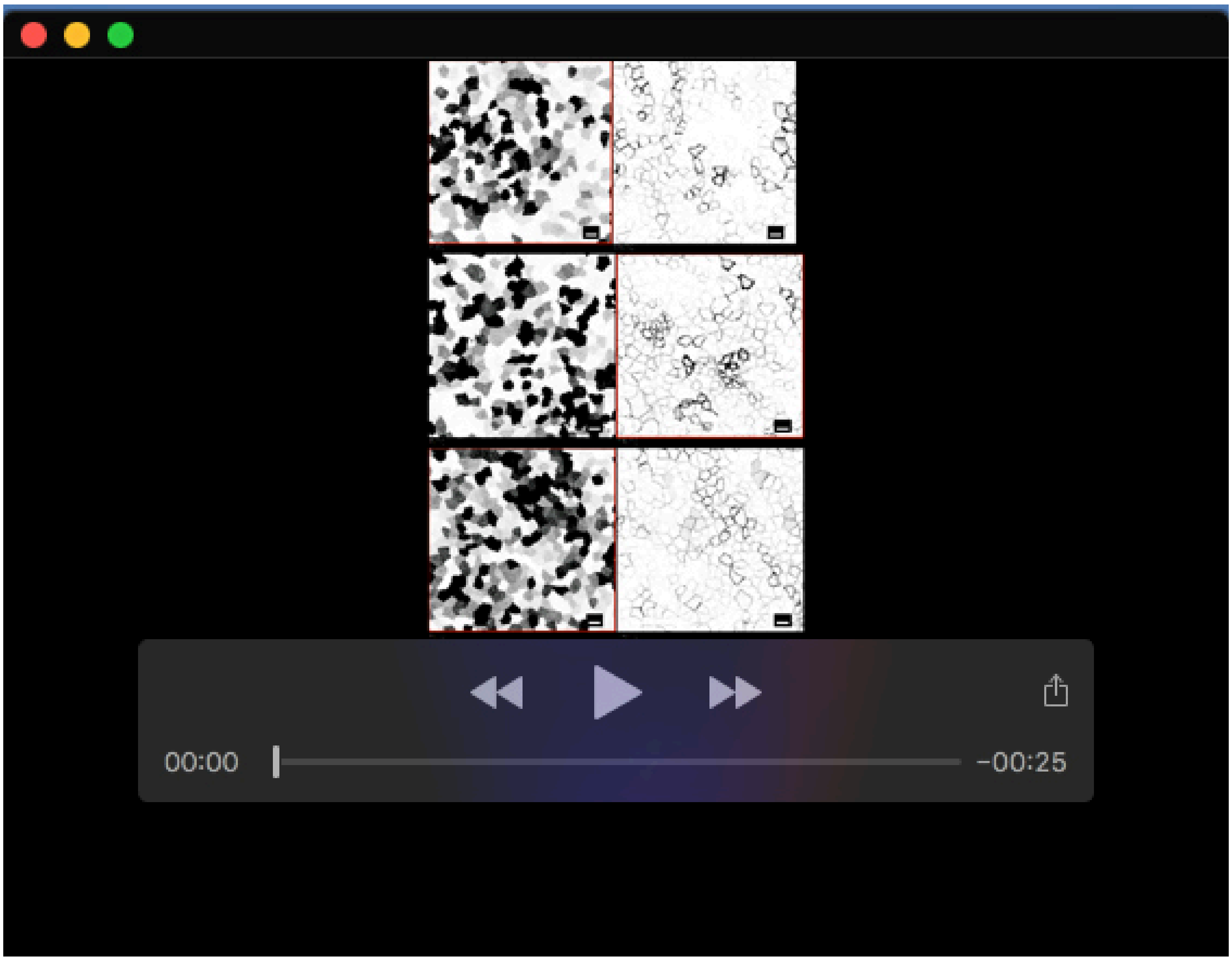

Movie 1. Internalization of E-cadherin-tdTom after induction of oncogenic Ras by + Dox. Ras expression is marked by GFP. Representative timelapse video.

Cells were imaged for up to 24hrs after Dox addition. Three independent fields of view are shown; the left panels display GFP fluorescence marking expression of Ras from the inducible GFP-P2A-Ras(Q61L) construct, and the right panels show E-cadherin-tdTom fluorescence. Images are maximum intensity projections from confocal slices generated on a Nikon A1R with 40x 1.2na oil objective. Note that GFP expression is not synchronized across the monolayers even though the cell line was clonally selected.

Journal of Cell Science • Supplementary information

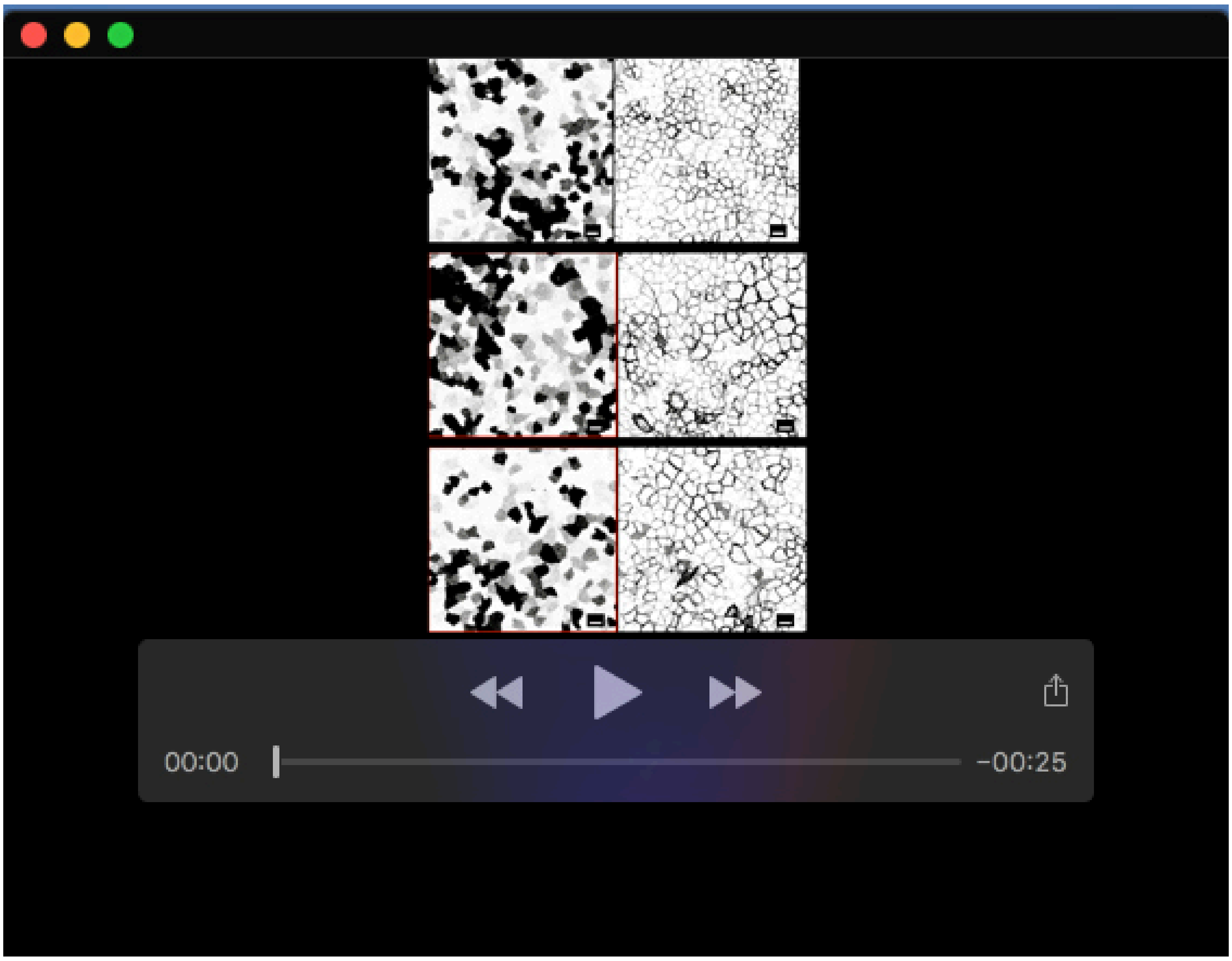

Movie 2. Representative video of the inhibition of E-cadherin-tdTom internalization by treatment with Erlotinib (10 µM). Ras expression is marked by GFP.

Cells were imaged for up to 24hrs after addition of Erlotinib and Dox. Three independent fields of view are shown; the left panels display GFP fluorescence marking expression of Ras from the inducible GFP-P2A-Ras(Q61L) construct, and the right panels show E-cadherin-tdTom fluorescence. Note that GFP expression is not synchronized across the monolayers even though the cell line was clonally selected.

Journal of Cell Science • Supplementary information
